# Supplementary material for: A burst-dependent thalamocortical substrate for perceptual awareness
Source: PLoS Comput Biol. 2025 Apr 7;21(4):e1012951. doi: 10.1371/journal.pcbi.1012951 (PMC12061433; doi:10.1371/journal.pcbi.1012951)
Supplement: S1 Appendix — Section A. Apical compartment phase plane. Fig A. A) Bifurcation diagram of the L5PT apical compartment. The saddle node bifurcation at IB1 generates a stable plateau potential which coexists with the resting state of the apical compartment until the model passes through a second saddle node bifurcation at IB2 at which point the resting state of the compartment vanishes and the plateau potential becomes globally attracting. B-C) Phase plane representation of the apical compartment showing the nullclines (black) for the following values of the bifurcation parameter; Iext < IB1, IB1 <Iext < IB2, Iext> IB2. Section B. Sweeping the magnitude of model perturbations. Fig B. A-C) Psychometric function fit to spiking model output across apical compartment excitation (blue), apical compartment inhibition (pink), and thalamic inhibition (orange), of varying magnitudes; A = 300 pA, B = 200 pA, C = 100 pA. D-F) Same as A-C but for neurometric functions; D = 300 pA, E = 200 pA, F = 100 pA. Section C. Robustness of rivalry duration across burstiness parameters. Fig C. Mean dominance duration as a function of the spike reset parameter values, and inter-compartment coupling probability, controlling the burstiness of the model L5PT cells. A) Dominance duration as a function of spike reset values with dynamic inter-compartment coupling (typical values are ~ 0.98 for the dominant population, and ~ 0.1 for the suppressed population; see Fig 3D). B – F) Dominance duration as a function of spike reset values with stationary coupling probability ranging from 1 to 0.6. Section D. Dynamical regime underlying visual rivalry. Fig D. A) Average firing rate of neuronal populations centred on opposite ends of the ring driven by a constant drive with asymmetric initial conditions. B) Average firing rate of neuronal populations simulated with constant drive and symmetric initial conditions. A perturbation was delivered at t=5000 ms sending the population orbit to the surrounding stable limit cycle [file pcbi.1012951.s001.docx]

**S1 Appendix:**

**A Burst-dependent Thalamocortical Substrate for Perceptual Awareness**

Christopher J. Whyte^1,2^, Eli J. Müller^1,2^, Jaan Aru^3^, Matthew Larkum^4,5^,

Yohan John^6^, Brandon R. Munn^1,2,#^, James M. Shine^1,2,#^

**Affiliations**

^1^Centre for Complex Systems, The University of Sydney, Sydney, Australia.

^2^Brain and Mind Center, The University of Sydney, Sydney, Australia.

^3^Computational Neuroscience Lab, University of Tartu, Tartu, Estonia.

^4^Institute for Biology, Humboldt University of Berlin, Berlin, Germany.

^5^NeuroCure Cluster of Excellence, Charité – Universitätsmedizin Berlin, Berlin,

Germany.

^6^Neural Systems Laboratory, Department of Health Sciences, Boston University,

Boston, MA, USA.

^#^Co-senior author.

***Section A. Apical compartment phase plane***

***
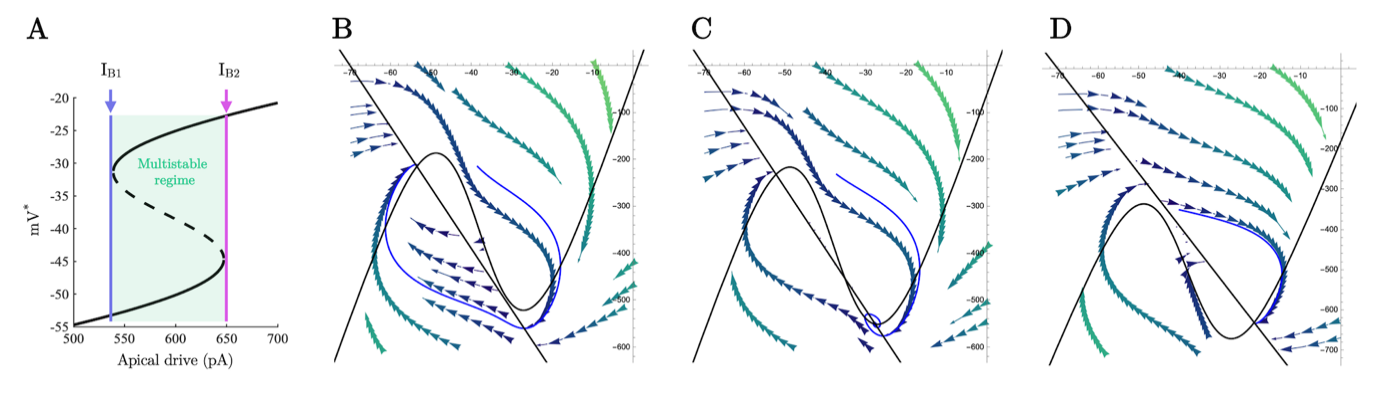
***

**Fig A. A)** Bifurcation diagram of the L5_PT_ apical compartment. The saddle node bifurcation at I_B1_ generates a stable plateau potential which coexists with the resting state of the apical compartment until the model passes through a second saddle node bifurcation at I_B2_ at which point the resting state of the compartment vanishes and the plateau potential becomes globally attracting. **B-C)** Phase plane representation of the apical compartment showing the nullclines (black) for the following values of the bifurcation parameter; $I_{ext}$< I_B1_, I_B1_ $<I_{ext}$< I_B2_, $I_{ext}>$I_B2._

***Section B. Sweeping the magnitude of model perturbations***

***
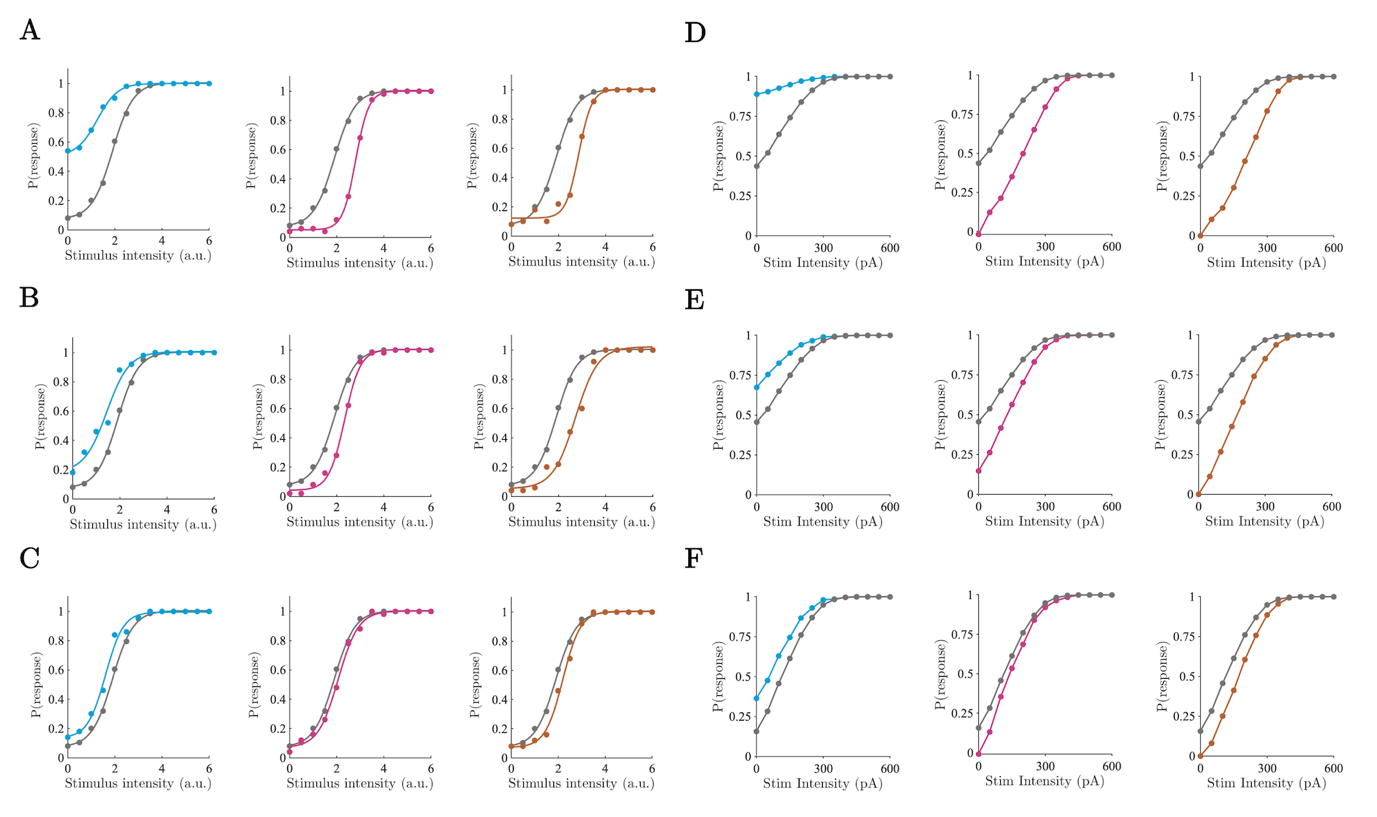
***

**Fig B. A-C)** Psychometric function fit to spiking model output across apical compartment excitation (blue), apical compartment inhibition (pink), and thalamic inhibition (orange), of varying magnitudes; **A** = 300 pA, **B** = 200 pA, **C** = 100 pA. **D-F)** Same as A-C but for neurometric functions; **D** = 300 pA, **E** = 200 pA, **F** = 100 pA.

***Section C. Robustness of rivalry duration across burstiness parameters***

***
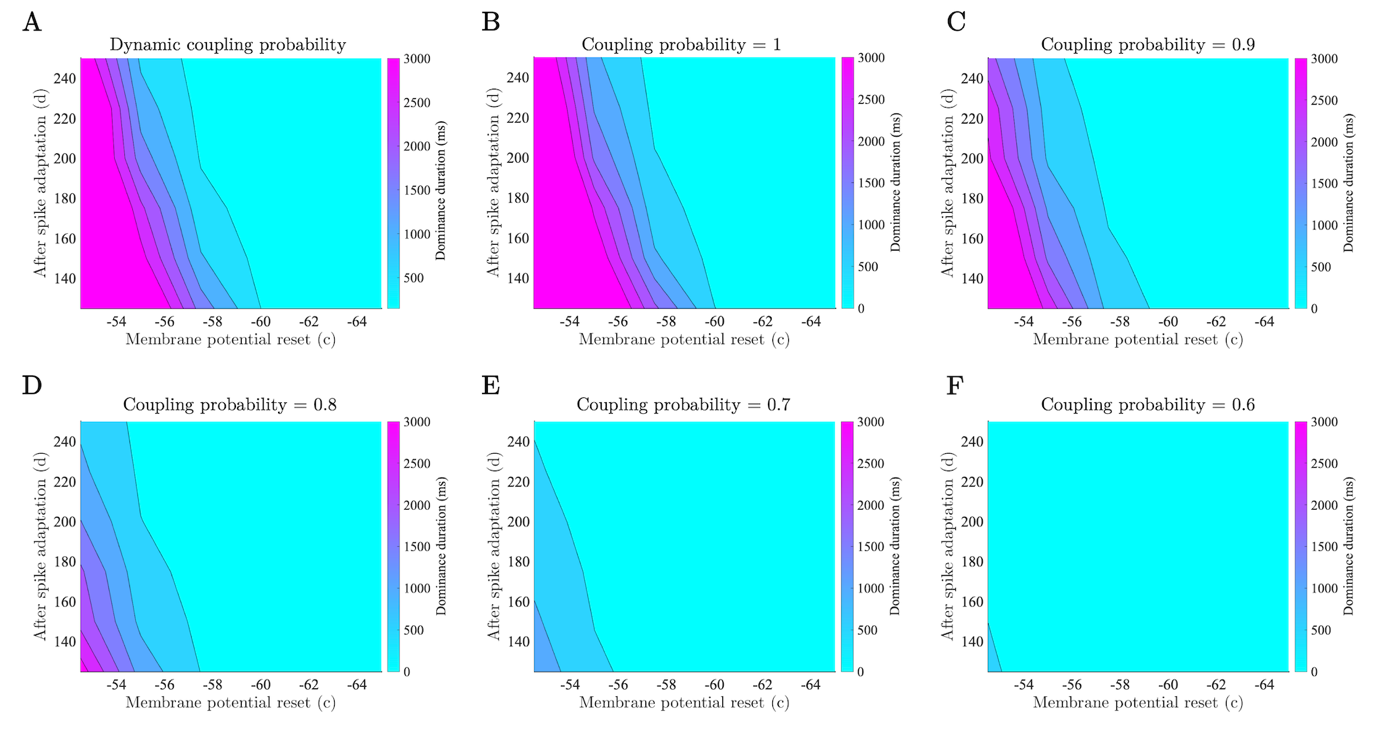
***

**Figure S3.** Mean dominance duration as a function of the spike reset parameter values, and inter-compartment coupling probability, controlling the burstiness of the model L5_PT_ cells. **A)** Dominance duration as a function of spike reset values with dynamic inter-compartment coupling (typical values are ~ 0.98 for the dominant population, and ~ 0.1 for the suppressed population; see **Fig 3D**). **B – F)** Dominance duration as a function of spike reset values with stationary coupling probability ranging from 1 to 0.6.

***Section D. Dynamical regime underlying visual rivalry***

To interrogate the structure of the dynamical system underlying the stochastic oscillations we made inter-compartment coupling deterministic and drove the model with a constant current and asymmetric initial conditions so that the system converged to a state where one of the populations was dominant whilst the other was suppressed. We reasoned that if the oscillations were driven by stochastic jumps between stable fixed points with basins of attraction modulated by adaptation then in the absence of noise the oscillations should disappear. In contrast, if adaptation exerts a large effect, the oscillations should consist of a stable limit cycle and the model should continue to oscillate in the absence of noise (c.f. (29,59)). In agreement with the stable limit cycle hypothesis in the absence of noise the model continued to oscillate (**Fig** **DA**). To test the stability of the limit cycle we: i) confirmed the existence of an unstable structure inside the limit cycle; and ii) confirmed that perturbations to the limit cycle decayed back to a stable orbit. With symmetric initial conditions the model converged to a state where excitatory activity on each side of the ring was perfectly matched. Perturbations to this state consisting of a 1 ms pulse of constant drive (50 [pA]) to the somatic compartment of a single L5_PT_ neuron caused the orbit to converge to the surrounding limit cycle (**Fig** **DB**). Such perturbations had little effect on already oscillating orbits confirming the stability of the limit cycle (**Fig** **DC**).


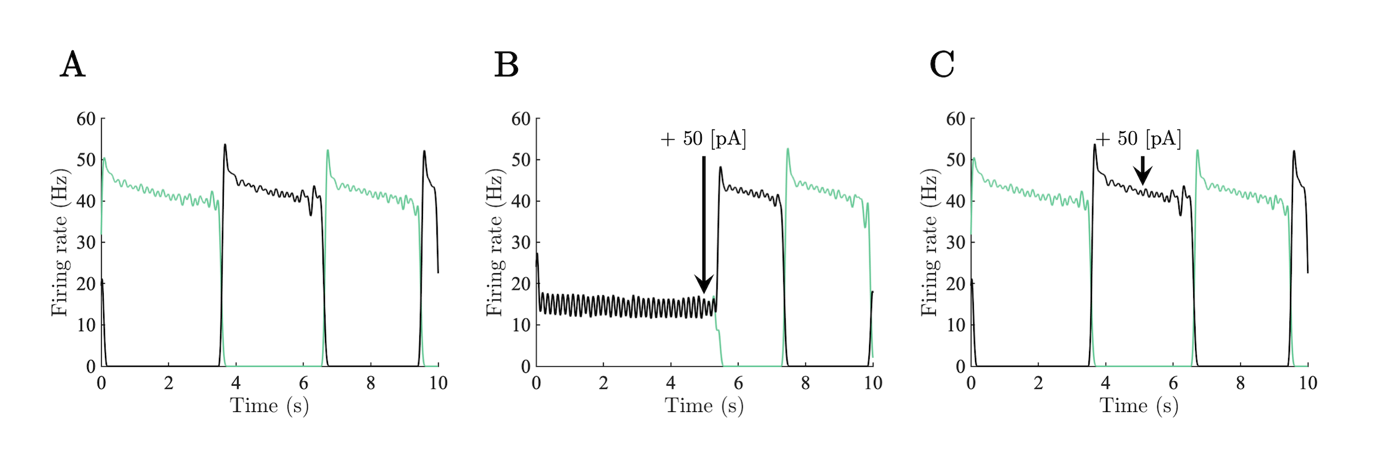


**Fig D. A)** Average firing rate of neuronal populations centred on opposite ends of the ring driven by a constant drive with asymmetric initial conditions. **B)** Average firing rate of neuronal populations simulated with constant drive and symmetric initial conditions. A perturbation was delivered at $t = 5000$ [ms] sending the population orbit to the surrounding stable limit cycle. **C)** Average firing rate of neuronal populations driven by constant drive with asymmetric initial conditions. Perturbation delivered at $t = 5000$ [ms] had no substantial effect on the already oscillating orbit indicative of a stable limit cycle.

***Section E. Key effects of visual rivalry simulations are preserved in scaled-up model***

To help guard against possible biases in the results caused by finite size effects or other simplifying assumptions made in the model such as the 50/50 excitatory/inhibitory neuron ratio, or the all-to-all connectivity of the cortical ring we constructed a scaled-up version of the model consisting of 2160 neurons (1600 excitatory, 400 inhibitory, 160 thalamic). The scaled-up model had sparse connectivity (12.5% connection probability), and an 80/20 excitatory/inhibitory neuron ratio (i.e. in line with Dale’s law). Because of the non-linearities in the model, and the reduction in the number of inhibitory neurons, we could not simply rescale the parameters of the original smaller network. Instead, we retuned the connectivity and adaptation parameters using the procedure described in **materials and methods**. Parameter values of the scaled-up network model are supplied below in **table S1 A**.

| **Parameter** | **Description** | **Value** | **Units** |
| --- | --- | --- | --- |
| $\lambda_{AMPA}^{E\to E}$ | Amplitude of excitatory to excitatory coupling for (AMPA) | $\frac{4.2}{\sigma^{E\to E} \sqrt{2\pi}}$ | a.u. |
| $\lambda_{NMDA}^{E\to E}$ | Amplitude of excitatory to excitatory coupling (NMDA) | $\frac{0.84}{\sigma^{E\to E} \sqrt{2\pi}}$ | a.u. |
| $\lambda^{E\to I}$ | Amplitude of excitatory to inhibitory coupling (NMDA and AMPA) | $\frac{1.5}{\sigma^{E\to I} \sqrt{2\pi}}$ | a.u. |
| $\lambda^{I\to E}$ | Amplitude of inhibitory to excitatory coupling (GABA_A_) | $\frac{5.85}{\sigma^{I\to E} \sqrt{2\pi}}$ | a.u. |
| $\lambda^{E\to TH}$ | Constant excitatory to thalamic coupling constant (AMPA only) | $4$ | a.u. |
| $\lambda_{AMPA}^{TH\to D}$ | Constant thalamic to apical dendrite coupling (AMPA) | $10$ | a.u. |
| $\lambda_{NMDA}^{TH\to D}$ | Constant thalamic to apical dendrite coupling (NMDA) | $10$ | a.u. |
| $\sigma^{E\to E}$ | Spread of excitatory to excitatory coupling | $0.25$ | a.u. |
| $\sigma^{E\to I}$ | Spread of excitatory to inhibitory coupling | $2$ | a.u. |
| $\sigma^{I\to E}$ | Spread of inhibitory to excitatory coupling | $2$ | a.u. |
| $\tau_{decay}$: AMPA | Decay time of AMPA conductance | $6$ | ms |
| $\tau_{decay}$: GABA_A_ | Decay time of GABA_A_ conductance | $6$ | ms |
| $\tau_{decay}$: NMDA | Decay time of NMDA conductance | $100$ | ms |
| $\tau_{coupling}$ | Decay time of apical coupling zone | $800$ | ms |
| $\tau_{adapt}$ | Decay time of adaptation current | 2000 | ms |
| $\Delta g$ | Contribution of each spike to adaptation current | 0.05 | nS |
| $\sigma^{VR}$ | Spatial spread of apical drive | 20 | a.u. |
| $E_{Excitatory}$ | Reverse potential of excitatory synapses | 0 | mV |
| $E_{Inhibitory}$ | Reverse potential of inhibitory synapses | -75 | mV |
| $E_{Adapt}$ | Reverse potential of adaptation currents. | -80 | mV |

**Table A.** Parameter description, values, and units of the (scaled-up) model components described by equations (5-11).

In line with the behaviour of the small network model reported in the main text, the large network model (**Fig EA**) generated a Gamma distribution of dominance durations (**Fig EB**), and was consistent with Levelt’s second (**Fig EC**) and fourth (**Fig ED**) propositions supporting the robustness of the burst-dependent mechanism of perceptual dominance put forward in the paper. All simulations run in the scaled up network lasted for 20 [s] and results were averaged over 20 random seeds.


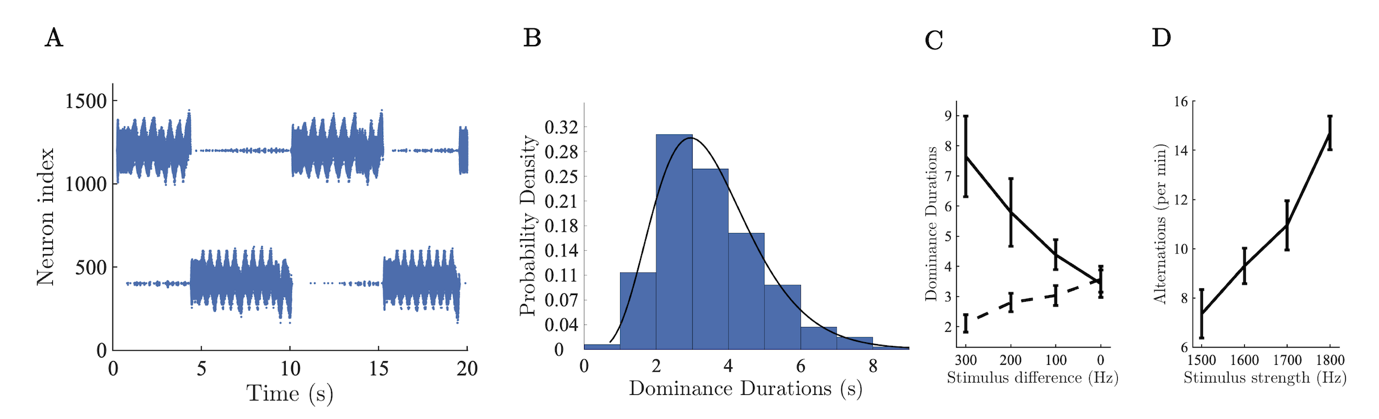


**Fig E. A)** Raster plots of somatic spikes from the scaled-up population of L5_PT_ cells **B)** Histogram of dominance durations, black line shows the fit of a Gamma distribution with parameters estimated via MLE ($\alpha$ = 6.2, $\theta$ = 0.56). **C)** Simulation confirming Levelt’s second proposition in scaled-up model. Dashed line shows the dominance duration of the population receiving the decreasing external drive, solid line shows dominance duration of population receiving a fixed drive. **D)** Simulation of Levelt’s fourth proposition in scaled-up model.

***Section F. In silico electrophysiology supplemental figures***


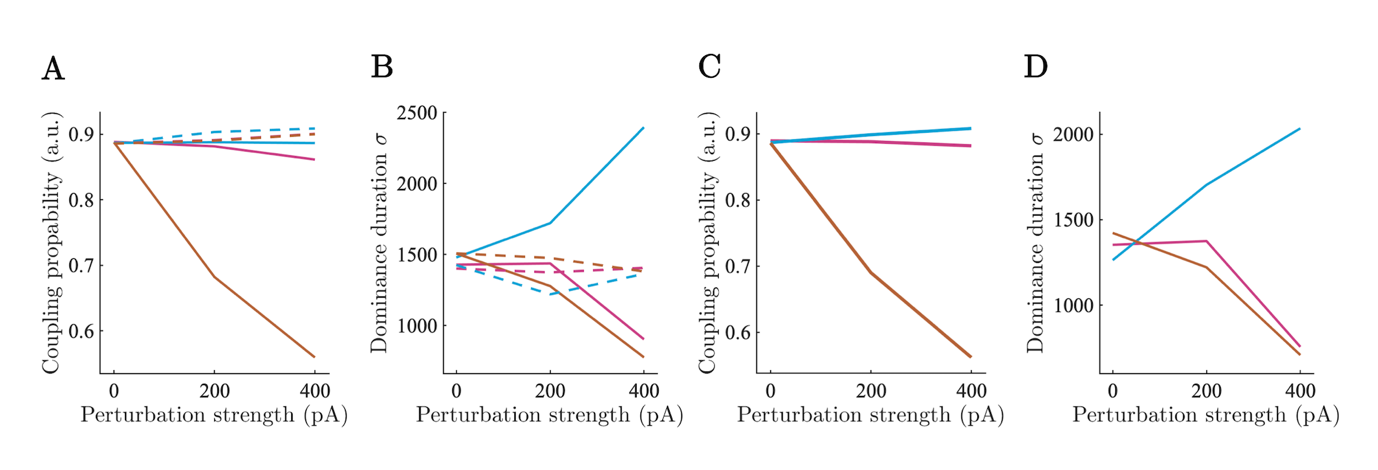


**Fig F. A)** Inter-compartment coupling probability under asymmetric perturbation for perturbed (solid) and unperturbed (dashed) populations as a function of perturbation strength for each perturbation type (colours same as main text). **B)** Dominance duration standard deviation under asymmetric perturbation as a function of the strength of each perturbation type. **C)** Inter-compartment coupling probability under symmetric perturbations. **D)** Dominance duration standard deviation under symmetric perturbation as a function of perturbation strength for each perturbation type.
