# Supplementary material for: A burst-dependent thalamocortical substrate for perceptual awareness
Source: PLoS Comput Biol. 2025 Apr 7;21(4):e1012951. doi: 10.1371/journal.pcbi.1012951 (PMC12061433; doi:10.1371/journal.pcbi.1012951)
Supplement: Attachment — Submitted filename: PCB_RR_Final.pdf [file pcbi.1012951.s003.pdf]

We thank the reviewers for their insightful and constructive comments which have substantially improved the quality of the paper.

Summary of revisions:

In the body of the paper, we provide a clearer neurobiological motivation for the more novel aspects of the circuity (i.e., matrix-thalamus mediation of the coupling between L5PT compartments), and using additional simulations (in supplementary material) have strengthened the link between the threshold detection and visual rivalry simulations. In line with reviewer recommendations, we also increased the range of the Levelt's law simulations and found that our model, in line with a number of well-established mean field models of rivalry, predicts a deviation from Levelt's fourth law for low values of external drive. In addition, to achieve a better comparison between the model's behaviour and empirical data, instead of using neurometric functions (which sum over all criterions), we now fit the same psychometric functions used in the empirical data to the output of the model. The neurometric function results have been moved to the supplementary material.

We have also added an additional three sections to the supplementary material addressing potential limitations of the model in which: 1) we show that the key visual rivalry results hold in a scaled-up version of the model that, in addition to being larger (helping to avoid potential finite size effects), also relaxes two of the less neurobiologically realistic assumptions made in the smaller network; 2) we sweep the parameters controlling the "burstiness" of the L5 PT cells and show that a minimum level of burstiness is necessary for the model to display visual rivalry with empirically realistic dominance directions, but that beyond this threshold the results are robust across a wide array of cellular parameter values; 3) we sweep the magnitude of the causal perturbations in the threshold detection simulations (simulated optogenetic excitation and pharmacological inhibition) and show that, as expected, the size of the effect varies with the magnitude of the perturbation, but crucially, the direction of the effect is preserved across perturbation magnitudes.

Full code necessary to reproduce all simulations reported in the manuscript can be found at: https://github.com/cjwhyte/LVPA

Below we respond in detail to each of the specific comments of each reviewer.

Reviewer 1:

The authors develop a semi-biophysical model for the interactions between layer 5 pyramidal neurons, inhibitory interneurons, and cells in the matrix thalamus. I cannot speak for the realism of the connectivity (I could find little in the literature about the

matrix thalamus that was not written by one of the authors of this paper; the little I did find suggested that the calbindin neurons (matrix) projected mostly to the superficial layers of the ctx, I, II)

Matrix-type thalamic cells are much less well studied by computational neuroscientists (but for notable exceptions see; Bonjean et al., 2012; Yazdanbakhsh et al., 2023) than traditional relay or core-type thalamocortical projections (i.e. driving projections to middle layers). Nevertheless, the core-matrix distinction is well-documented in neuroanatomical and neurobiological literatures. The distinction was initially proposed by Ted Jones based upon non-human primate histology (Jones, 2001, 2002; Jones & Hendry, 1989). Broadly speaking, calbindin staining (matrix-type) cells have dense but diffuse projects across superficial layers (L1/2) and sparse (but no less important) projections to L5a. Parvalbumin positive core-type cells have comparatively precise projections to L3/4, as well as L6 (for a review of recent evidence see Shepherd & Yamawaki, 2021). The initial findings in non-human primates were subsequently found to hold in both post-mortem human tissue (Münkle et al., 2000), and mice (for review see Clascá et al., 2016; Shepherd & Yamawaki, 2021). Most recently, the distinction has been championed and extended by Clascá and colleagues who subdivide the matrix-type cells into M-type multiareal cells which have diffuse projections across L1/2 and sparse projections to L5a, and M-type focal projections which have targeted projections to L1/2 (Clascá et al., 2012). Based on the work of Suzuki and Larkum (2020) showing the importance of thalamic projections to metabotropic receptors on the oblique dendrites of PT type cells in L5a we chose to include the M-type multiareal cells in the model.

The paper seems more like two papers. One would be very short and concerns the responses of layer V neurons in the barrel cortex of mice to whisker flicks and how they are modulated by optogenetic and pharmacological manipulation. Again, the circuitry here surprised me as my understanding was that the flick of the whisker activated VPM which then feeds directly forward to layer IV of the barrel cortex, so i wonder what happened to that part of the circuit. I also thought layer IV -> II/III which interacts with V, so how does one eliminate that major circuit?

Re whether to split our paper into two: Given the fact that the empirical results have already been published in two different manuscripts (Takahashi et al., 2016; 2020), and that our expressed goal was to build a minimal circuit model that included only the neuronal elements necessary to reproduce the animal's behaviour in the experiments carried out in (Suzuki & Larkum, 2020; Takahashi et al., 2016a, 2020) that we could then extend to generate novel predictions it would not make sense to split the paper into two.

Relatedly on the questing of what circuitry to include, we elected to build a minimal model including only the minimum number of circuit elements necessary to reproduce the experimental data we were able to ensure the interpretability of our model. We, therefore, only included L5 PT-type cells (optogenetic manipulations of L5 IT type cells was found to have no effect on the mouse's perceptual threshold), inhibitory interneurons (basket cells) which target the somatic compartment of L5

PT cells (Harris & Shepherd, 2015) ensuring E/I balance, and matrix-type thalamic cells. We have no doubt that other elements in the circuit play an important role, but because they were not directly targeted experimentally, we could get away with modelling them implicitly. For example, FF projections from VPM to L4 are implicit in the excitatory (Poisson) drive hitting the somatic compartment of our L5 PT type cells (L4 IT cells have excitatory projections to the somatic compartment of L5 PTs; Harris & Shepherd, 2015). L2/3 cells likewise no-doubt play a crucial role in the function of the cortical column but as they were not directed manipulated experimentally we did not include them explicitly leaving their dynamics implicit in the recurrent E to E connections. This is included as a notable limitation in the discussion section: (P.24): "*Most notably, we did not include a L2/3 pyramidal neuron population – which are the primary source of long distance horizontal connections in the cortex (Douglas & Martin, 2004) and arguably cross column inhibition (Qian et al., 2023)...*"

Re the question of why we chose to model both whisker deflection and visual rivalry in one model: Matrix thalamus mediated bursting in L5 PT cells has been hypothesised to be a potentially necessary condition for sensory contents to become conscious (Aru et al., 2019, 2020; Storm et al., 2024), but experiments have so far not extended beyond simple threshold detection paradigms. We aimed therefore to build a model that could: 1) reproduce and explain the initial threshold detection findings, and 2) be extended to simulate other more sophisticated experimental paradigms used to study perceptual awareness allowing us to generate additional empirical predictions. We chose visual rivalry (which we use as a catch all term for binocular rivalry and related bistable perception paradigms such as plaid perception), as it is very well studied imposing a suit of constraints on the modelling process. In addition, because plaid perception can be studied in mouse models (Bogatova et al., 2024; Palagina et al., 2017) all of the same experimental manipulations (optogenetic and pharmacological) carried out in the original whisker deflection experiments could (in principle) be carried out in the context of visual rivalry allowing us to generate a series of testable predictions.

The part of the paper on rivalry (BR) doesn't really distinguish itself from other models although, in its favor, it provides some experimental predictions. However, I would like to see more details on what BR experiments can be done on mice. Plaids are mentioned, but no details are given. I really don't see any easy way to tie these two parts of the paper together. The ring structure seems to be irrelevant to the whisker flicking and pretty much any network with a ring structure and long range inhibition will give rise to WTA type dynamics. Since the Py cells have adaptation, this is basically the same model as others have proposed. The authors make a big deal about the importance of the bursting properties of the Py cells but it is unclear why that is important for BR. I don't know much about the thalamic matrix, but in the other sensory thalami, the TC cells have a prominent T-type calcium current and thus are capable (and in fact do so routinely) of producing bursts. They are also subject to modulation that takes them from burst to relay mode; there is a vast and old literature on this as well as models going back at least to the 90's. There are also reticular inhibitory neurons which seem to be missing in the model. Perhaps they are not in the matrix thalamus.

Re: visual rivalry in mice: Plaid perception, is used in the mouse model, instead of binocular rivalry, as mice have very little binocular overlap in their visual field (due to the lateral positioning of their eyes; Poort & Meyer, 2021). Motion-rivalling plaid stimuli can be delivered to one eye eliminating this issue. The dominant percept is then inferred using eye-tracking, involuntary eye movements (optokinetic nystagmus) track the direction of the dominant percept, not the suppressed percept and can therefore be used to indirectly infer the contents of perception. Crucially, plaid perception still obeys the same law-like psychophysical constraints (Levelt's laws; Brascamp et al., 2015; Hupé et al., 2019) as binocular rivalry, and also has a Gamma distribution of dominance durations (Bogatova et al., 2024). Therefore, although it is undeniably an inference that experimental manipulations of plaid perception in the mouse will be governed by similar mechanisms as binocular rivalry in primates we believe this inference is well justified.

We have included the following text in the manuscript (P.15): *"Binocular rivalry is thought to depend in part on the substantial degree of binocular overlap in humans (~120°), however the lateral position of the eyes in mice leaves only ~40° of binocular overlap (Poort & Meyer, 2021). For this reason, there are no current mouse models of binocular rivalry, however there are monocular variants of visual rivalry, namely plaid perception, that can be studied the mouse model (Bogatova et al., 2024; Palagina et al., 2017). Crucially, plaid perception, like binocular rivalry, conforms to Levelt's laws (Brascamp et al., 2015; Hupé et al., 2019) and also has a right skewed distribution of dominance durations that is well fit by a Gamma distribution (Bogatova et al., 2024). We hypothesise, therefore, that the principles underlying the simulation of binocular rivalry in our model will also describe other forms of visual rivalry (such as plaid perception), offering a plausible means to test cellular level predictions derived through simulation."*

Re distinguishing our model from others in the literature: We did not aim for our model to displace any of the existing models of rivalry in the literature. Rather, instead of characterising the minimal neuronal conditions necessary for rivalry which is the target of explanation for many, if not most, model of rivalry, we aimed to test the hypothesis that matrix-thalamus mediated bursting in L5 PT cells is the basis of perceptual dominance *in silico*. Therefore, as the reviewer rightfully points out, we provide predictions about neuronal level circuit manipulations which would not be possible to model in any existing model. To make this point clear to readers following text is included in the discussion:

(P.22) "Importantly, the simulation of these experimental perturbations would not be possible in any existing models of rivalry, even those at the spiking level (e.g. Laing & Chow, 2002; Wang et al., 2020; Wilson, 2003), as they focus on the minimal conditions for rivalry in point-neuron models of cortical interaction. The inclusion of a dual compartment model of $L5_{PT}$ cells, and an explicit thalamic population, was, therefore, required in order to make contact with the results of Takahashi et al (2016, 2020)."

Re matrix thalamus and bursting: We agree that the classic T-type calcium current inhibition induced / rebound bursting results in part form interactions with the RTN, and plays an important role in the generation of sleep spindles, and slow waves in sleep. Crucially, however, we are not aiming to model thalamic bursts. Rather we aimed to model the matrix thalamus mediation of intrinsic bursting in cortical L5 PT cells. Specifically, bursting in L5 PT cells relies on reliable coupling between the soma and apical dendrites allowing sodium spikes to back propagate and contribute to the generation of a calcium spike in the apical dendrites, and in turn, for calcium spikes in the apical dendrites to propagate down the trunk of the cell to the soma and contribute to the generation of a burst of sodium spikes. Communication between these compartments relies upon depolarising input from matrix thalamus cells to metabotropic receptors on the distal dendrites of L5 PTs located in L5a (Suzuki & Larkum, 2020). As all of our simulations were aimed at explaining the behaviour of mice in the waking state, the thalamus is in a tonic state, and were therefore did not explicitly model the RTN population.

Re connecting the threshold detection and rivalry simulations. As we discussed above, our aim was to explore the hypothesis that matrix thalamus mediated bursting in L5 PT cells is a necessary condition for perceptual awareness, and therefore, should also underlie perceptual dominance in visual rivalry. In the simulations we report, we explicitly simulate all of the same experimental manipulations performed in the context of threshold detection in visual rivalry highlighting the shared nature of the underlying mechanism. In addition, we have now included a sweep of the cellular level parameters of the spiking model which underlie the thresholded detection effects in supplementary S3. We show that the existence of dominance durations in an empirically plausible range requires a minimum level of burstiness further reinforcing the mechanistic link between the threshold detection and visual rivalry simulations.

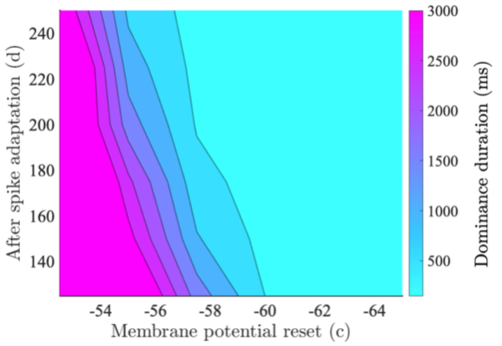

Figure S3. Mean dominance duration as a function of reset parameter values which control the burstiness of the model $L5_{PT}$ cells.

Other points:

page 9: is there a reason the thalamic projection don't synapse the inhibitory neurons? That is certainly the case in layer IV

Yes this because the (multiareal) matrix-type projections hit superficial layers and layer 5a. There is some evidence suggesting that these cells innervate neurogliaform cells in superficial layers (Schulz et al., 2021) but there is a lot of uncertainty in the connectivity thus for simplicity we kept this element out of our model.

Fig 1B: the firing patterns of the neurons -- are they all synoptically coupled in the network? Why does one excitatory spike lead to a long burst of activity in the inhibitory neuron? Is there some long lasting synapse?

Yes they are synaptically coupled. We have made a note of this in the figure caption for clarity.

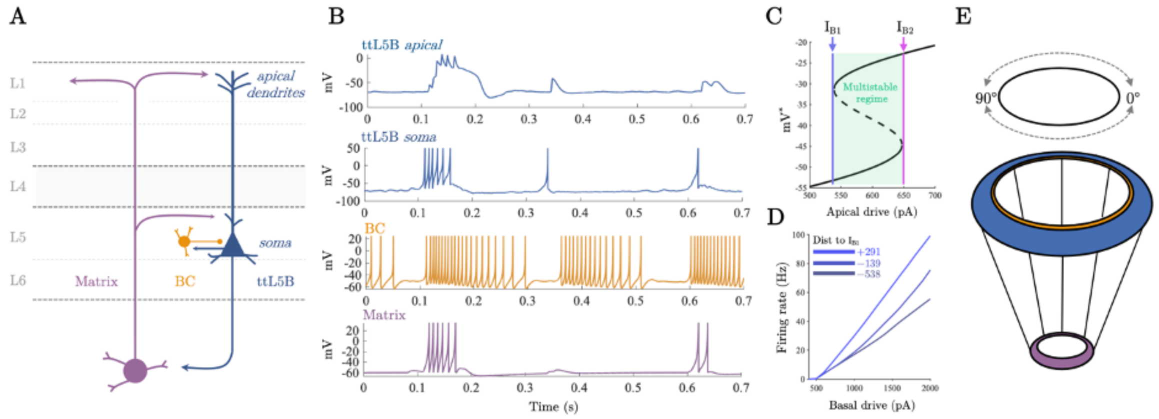

Fig 1. Thalamocortical model of perceptual awareness. A) Idealised anatomy of the model thalamocortical loop connecting higher-order matrix thalamus and L5PT neurons. **B)** Single neuron dynamics of example neurons in the thalamocortical network for each class of cell when driven with 600 [Hz] of independent background drive to the somatic compartment of every neuron and 50 [Hz] to apical compartment of L5PT cells. **C)** Bifurcation diagram of the L5PT apical compartment. I_{B1} denotes a saddle node bifurcation generating a stable plateau potential which coexists with the resting state of the apical compartment until the model passes through a second saddle node bifurcation at I_{B2} at which point the resting state vanishes and the stable plateau potential becomes globally attracting. **D)** Somatic firing rate of the novel dual compartment L5PT model as a function of basal (i.e. somatic compartment) drive and apical drive (measured in terms of the distance to I_{B1}). In line with empirical data (Larkum, 2004),

page 12: The data in fig 2c seem to show a much sharper threshold than the model in the control case. Is there a reason for this? Why are the data controls in c and d so completely different? The thresholds are also very different. Not sure how much credence to put in the data if the controls are so different despite being under identical circumstances

2c and 2d represent data from very different experiments. In c we have an experiment with baclofen, whereas in d, the experiment is done with optogenetics. Different mice were used for these experiments. Variance between mice is very common. The effect of optogenetic excitation of L5 PT apical dendrites on perceptual thresholds has been replicated in two separate experiments.

On the difference between model and data: this is due to the use of neurometric functions (which average over all criterions) in the model, whereas psychometric functions were fit to the empirical data. To address this issue, in the revised manuscript we now fit the same psychometric function used by (Takahashi et al., 2016b, 2020) under the assumption that the model uses the optimal criterion (the criterion that best minimises misses and false alarms). This new method results in a sharper perceptual threshold more like the empirical data.

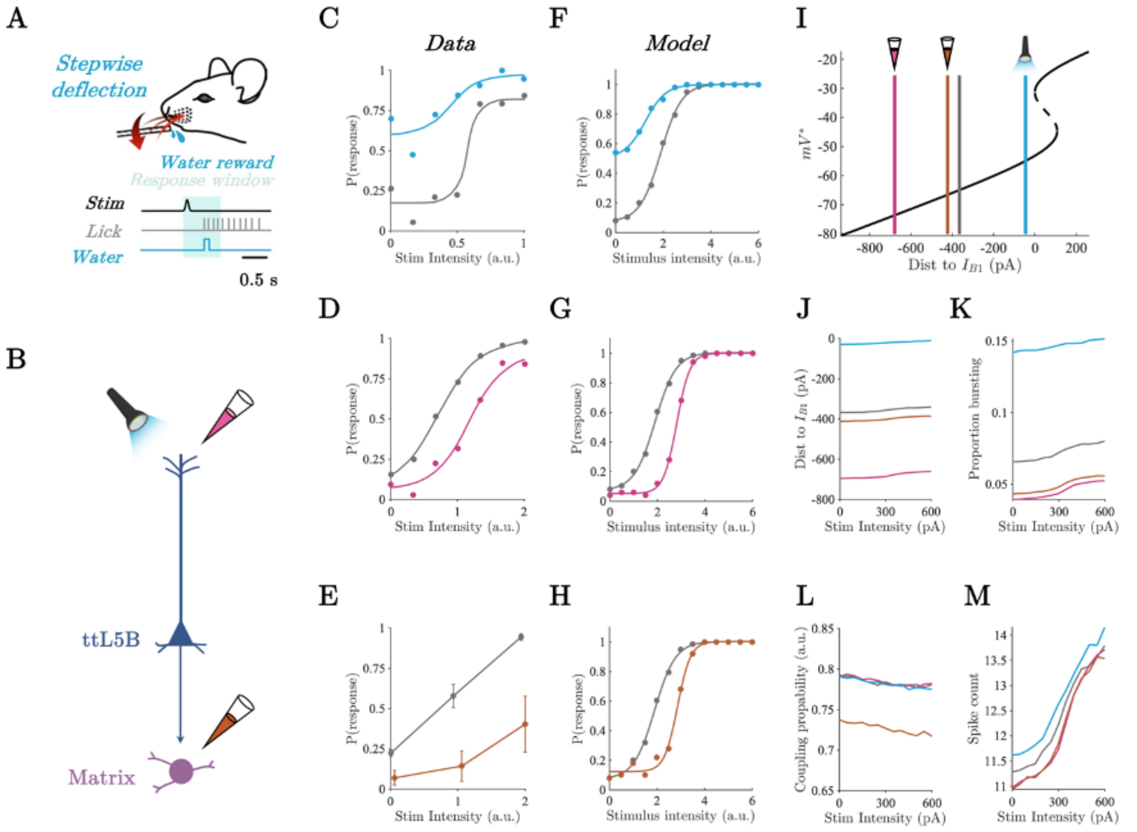

Fig 2. Apical compartment distance to bifurcation and thalamic-gating explains shifts in perceptual threshold. A) Whisker deflection paradigm modified from Takahashi et al, (2020). **B)** Representation of causal perturbations to L5PT thalamocortical circuit including optogenetic excitation of the apical dendrites (light blue), pharmacological inhibition of the apical dendrites (pink), and pharmacological

page 14: Distance to bifurcation seems to be a major part of the paper. How did you compute this distance from the fold? The model is stochastic so it is not clear to me how you get such precision in distance metrics. I understand how you find the folds as that is just algebra, but how do you get the values in fig 2I

We are looking at the average distance to bifurcation in the apical compartment across the population in the post stimulus window (i.e. we approximate the current entering the apical compartment with the average value of the synaptic drive across the population). We make now make clearer in the manuscript that we are looking at the average distance to bifurcation across the population (P.10): *"Excitation of*

the apical dendrites (blue) reduced the average distance to bifurcation (here defined as the distance to I_{B1}) in the apical compartment across the network in the 1000 [ms] period post stimulus onset"

How was pharmacological inhibition mediated in the model? How do you know how strong to make it? Do you just change it until the model matches the data?

We ran sweeps across a range of values all of which were relatively consistent. These are now noted in the text and included in supplementary material S2 for by psychometric and neurometric functions.

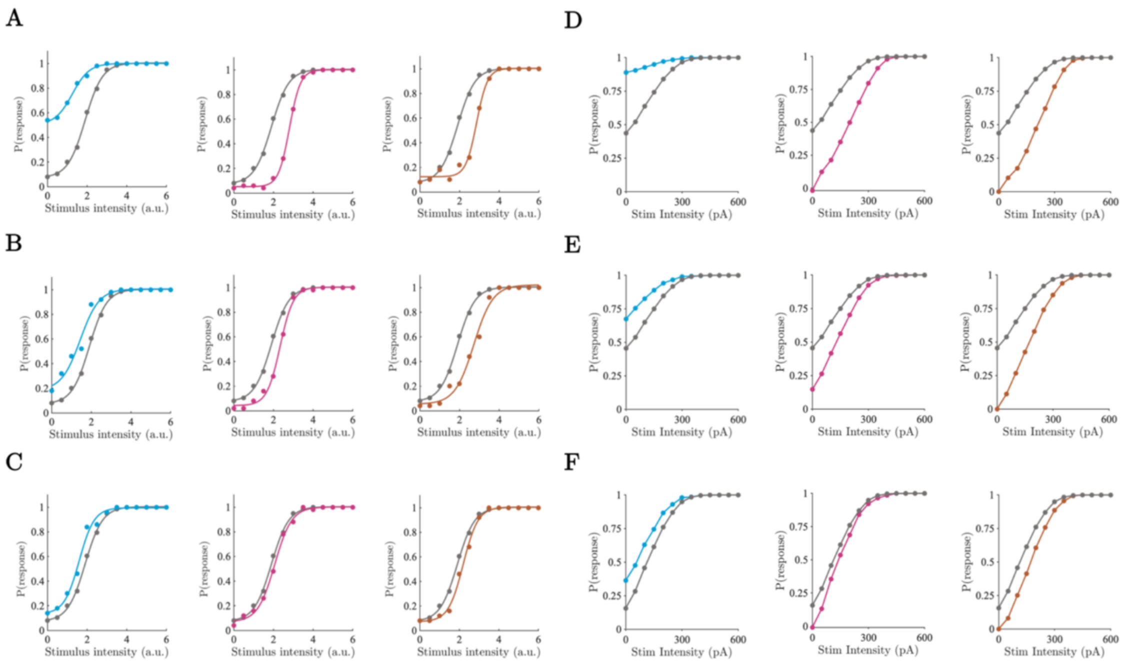

Figure S2. A-C) Psychometric function fit to spiking model output across apical compartment excitation (blue), apical compartment inhibition (pink), and thalamic inhibition (orange), of varying magnitudes; **A** = 300 pA, **B** = 200 pA, **C** = 100 pA. **D-F)** Same as A-C but for neurometric functions; **D** = 300 pA, **E** = 200 pA, **F** = 100 pA.

page 16: In 3 D, what are the black curves?

The black curves represent values of the initially dominant population that becomes suppressed after the perceptual switch, coloured curves represent values of the initially suppressed population that is dominant after the switch this is now better highlighted in figure caption.

page 17: The whole bit about analytic proofs of limit cycles is bizarre and unnecessary. This is not really relevant as your odes are intrinsically stochastic in that there is a probability of switching to bursting, and it is not my impression that you turned that "noise" off. In a purely deterministic model, the hopf theorem provides a way to prove existence of limit cycles in any dimension. This whole section should probably be rewritten or eliminated

We agree that the Hopf theorem provides a way to prove the existence of a non-trivial periodic orbit for continuous deterministic systems. However, we cannot use the Hopf theorem as our spiking model has a discontinuity violating the assumption of a smooth vector field. Instead we opted to use a heuristic line of argument based on simulations in the absence of noise by switching the input to a constant current and making bursting deterministic instead of probabilistic. As requested we have rewritten the section and noted that bursting is deterministic in the supplementary material where the simulations are discussed in detail.

(P.13): "Because of the large size of the network and the discontinuity in the somatic compartment we could not use analytic methods to interrogate the structure of the dynamical system underlying the stochastic oscillations. Instead we used a heuristic line of argument (see Strogatz, 2018 Ch.8) combined with simulations in the absence of noise to confirm that the dynamical regime underlying these stochastic oscillations likely consists of noisy excursions around a stable closed orbit (i.e. a stable limit cycle; supplementary material S2)."

(P.33): "To interrogate the structure of the dynamical system underlying the stochastic oscillations we made inter-compartment coupling deterministic and drove the model with a constant current and asymmetric initial conditions so that the system converged to a state where one of the populations was dominant whilst the other was suppressed...."

page 18: What happens with continued lowering of the input strengths? In the models that were collected in Shpiro et al, they find that the period is non monotone with respect to strength. I'd be interested to see what happens as the stimulus decreases. In most cases there are hopf bifurcations at each end.

When input strength is lowered our model, like the models explored in (Shpiro et al., 2007), show a reversal of Levelt's fourth law for low stimulus strength values. We have now included simulations across a wider range of values to highlight the presence of this behaviour in our model (Fig 2G shown belw).

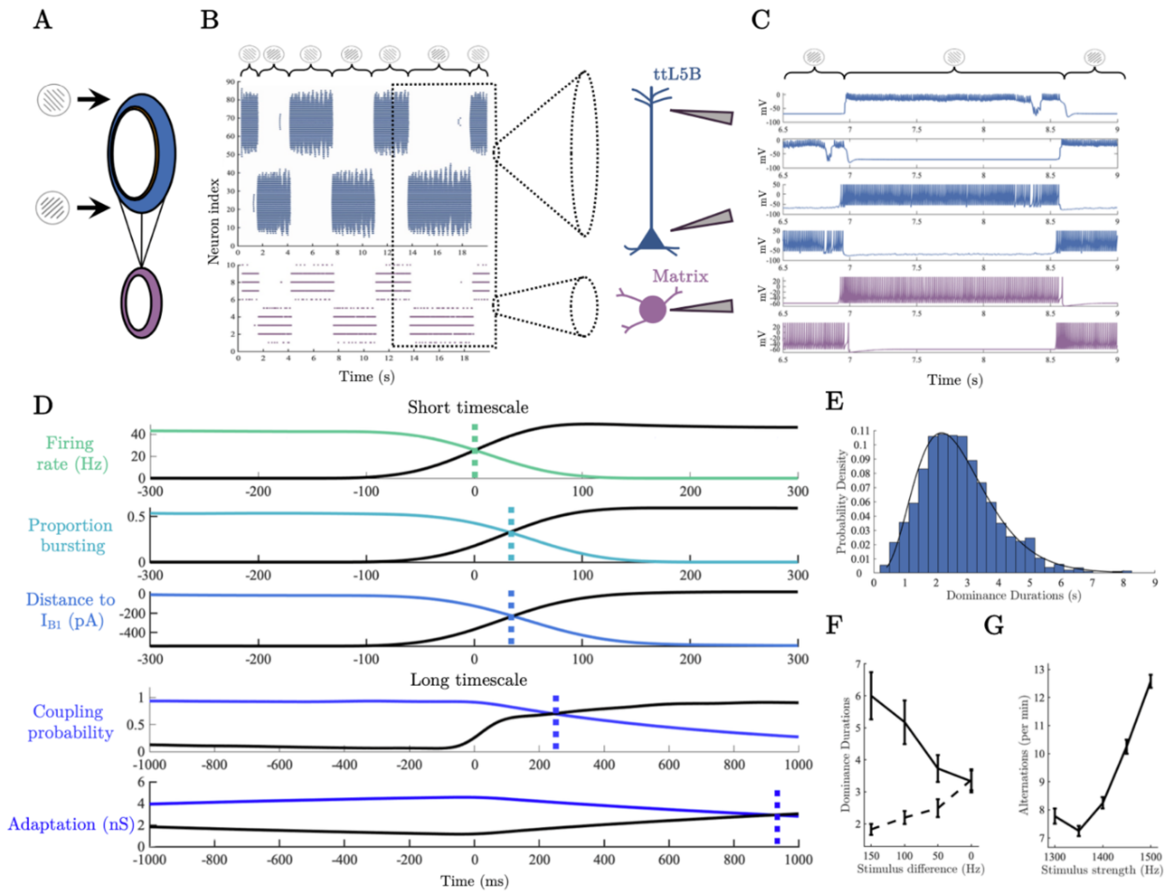

Fig 3. A thalamocortical cascade underlies perceptual switches. A) Thalamocortical ring architecture driven by input representing orthogonal gratings. **B)** Raster plots of $L5_{PT}$ soma (blue) and matrix thalamus population (purple) during rivalry. **C)** Example single neuron spiking activity around a perceptual switch – dotted lines denote the crossing in the population averaged time series. **D)** Population averaged neuronal variables centred on a perceptual switch. **E)** Histogram of dominance durations, black line shows the fit of a Gamma distribution with parameters estimated via MLE (α = 3.7863, θ = 0.6272). **F)** Simulation confirming Levelt's second proposition. Dashed line shows the dominance duration of the population receiving the decreasing external drive, solid line shows dominance duration of population receiving a fixed drive. **G)** Simulation of Levelt's fourth proposition. Notably, for low stimulus strengths our model, along with a number of meanfield models of visual rivalry (Shpiro et al., 2007), predicts a deviation from Levelt's fourth proposition.

page 20: What does perturbation strength mean for unperturbed conditions? Why isn't it flat ?

This refers to the population in the network that is perturbed in the asymmetric perturbation condition where we only inhibit/excite one of the competing dendritic populations and leave the other untouched. We have made this clearer in the figure caption.

P16: "Causal perturbations to one half of the thalamocortical circuit underlying visual rivalry consisting of optogenetic excitation of the apical compartment (blue), pharmacological inhibition of the apical compartment (pink), and pharmacological inhibition of the thalamus (orange)"

page 30: you use f(x) and f(s) for different things. Change symbols

We now use f(x) to denote the membrane potential non-linearity and have changed f(s) which we previously used to denote the occurrence of a spike in the somatic compartment with t^s in line with the notation used throughout the rest of the paper.

Reviewer 2:

This manuscript presents a computational model of thalamocortical networks which is able to explain recent experimental findings regarding the role of dendro-somatic communication of layer 5 pyramidal cells in perceptual awareness. The authors then take their biophysically realistic model and use it to explore experimental findings on visual rivalry, thus extending the original claims of the experimental papers towards other tasks and sensory modalities, replicating classical results such as Levelt's binocular rivalry propositions, and providing new testable predictions.

The topic is timely and relevant, the work is carried out diligently, and the results will certainly be of interest for the computational neuroscience community, as well as for researchers in perception and awareness topics. The current version however contains a few limitations and modeling choices that might need to be reassessed in order to be fully convincing. These issues are summarized below.

1) A network of 180 neurons is definitely on the lower side in terms of network size. Spiking networks that small might suffer from finite size effects and provide misleading results. This is even worse for the present case, as the orientation selectivity ring structure means that each window of 2 degrees will only have one excitatory neuron representing it. In real neural circuits, we would expect to have at least a small population for each (approximate) orientation. Given that the authors use Izhikevich models for their neurons, which is a highly efficient model computationally, it should not we a problem to go into a more reasonable size of 5,000-10,000 neurons, so that each box of 2 degrees is encoded by approximately 50-100 neurons.

2) The cortical ring seems to have an equal number (90) of excitatory and inhibitory cells. This is a strange choice given that the E:I ratio in most cortical regions is about 80:20. A larger number of excitatory cells, especially when recurrently connected (see point below) could lead to destabilizing the system's activity or change the bursting patterns, and therefore it's important that the authors show whether their main findings hold for this more realistic ratio.

3) Related to the above two points, simulating a larger network would permit the existence of recurrent connectivity between each pool of 50-100 L5 pyramidal cells encoding the same orientation, which is something expected from a realistic model.

The absence of recurrent connectivity could hinder potential problems with the model, such as pathological synchrony or hyperexcitability. A supplementary study showing the impact of such recurrent connections could be done to dissipate doubts.

We very much agree with concern about the potential presence of finite-size effects in the model. To address this issue we have added a section to the supplementary material (S5) showing that the key simulation results hold in a scaled-up version of the model that respects Dale's law. We note, however, that although Izhikevich neurons are computationally efficient our model is considerably more complicated than typical models built with Izhikevich neurons. For instance, we use conductance based synapses with three different receptor types and have separate synapses for the targets of FF drive and recurrent drive, we include separate compartments for the soma and apical dendrites, we have an additional equation representing the slow hyperpolarising current, and have an additional conductance based term governing the coupling between the compartments. Each excitatory cell is, therefore, governed by 9 ODEs instead of the usual 2. With these constraints, even the relatively small 180 cortical neuron network requires 1630 ODEs. In addition, because we are simulating psychophysical effects we need to run very long simulations (~45 minutes of neuronal activity per simulation), which with an integration step of 0.1 ms, is much more computationally intensive than standard biophysical simulations.

With this in mind, we chose to scale up to a network of 2000 cortical neurons (2160 including thalamus, implemented in 22880 ODEs). To address the concern about Dale's law we chose an 80:20 ratio of excitatory to inhibitory neurons (1600 excitatory neurons, 400 inhibitory neurons, and 160 matrix thalamus neurons). We also took the opportunity to relax the assumption of all-to-all connectivity. We gave each cortical neuron a 12.5% probability of connecting to another cortical neuron. Crucially, simulations in the scaled up model still obeyed Levelt's second and fourth laws (the others laws are a logical consequence of these and do not need to be explicitly simulated) and produced a Gamma distribution of dominance durations.

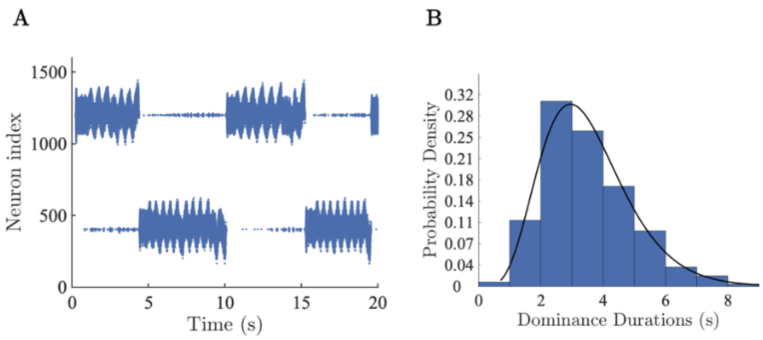

Figure S5. A) Raster plots of somatic spikes from scaled population of L5PT cells **B)** Histogram of dominance durations, black line shows the fit of a Gamma distribution with parameters estimated via MLE ($\alpha = 6.2$, $\theta = 0.56$). **C)** Simulation confirming Levelt's second proposition in scaled up model. Dashed line shows the dominance duration of the population receiving the decreasing external drive, solid line shows dominance duration of population receiving a fixed drive. **D)** Simulation of Levelt's fourth proposition in scaled up model.

4) The model keeps the same parameters to simulate the tactile threshold detection as well as visual rivalry tasks. While I appreciate and agree with the idea that showing a generic model perform these tasks is more convincing than having individually tuned models, it is also true that circuit properties might substantially vary across species. Cortical pyramidal cells, in particular, are known to display substantial differences between mice and primates (Gilman et al. Cereb. Cortex 2017, Mihaljevic et al. Front. Neuroinform. 2021, Kalmbach et al. Neuron 2018 & 2021). Therefore, it is possible that some of the results might be substantially vary across species and the model might be missing this fact by committing to only a given parameter set. One way to solve this would be to show how robust the main results are when certain parameters are varied in the model, to account for species-related differences in cell physiology. This could be shown in a supplementary figure, for example.

This is an excellent point. We have added an additional section to the supplementary material (S3) sweeping the c and d parameters which control the "burstiness" of model L5 PT cells. The presence of rivalry with empirically realistic durations between populations requires that the L5 PT cells have a minimum level of "burstiness", but beyond this threshold rivalry is robust across a range of parameters.

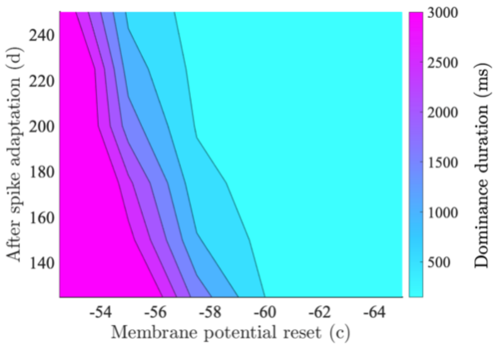

Figure S3. Mean dominance duration as a function of reset parameter values which control the burstiness of the model $L5_{PT}$ cells.

5) Fig 2 seems to show that model predictions regarding the upwards shift of the psychometric functions tend to underestimate the magnitude of the shift for strong inputs. Would this be a limitation from the data (i.e. experiments not going for strong enough inputs) or from the model (i.e. absence of adaptation mechanisms for strong input)? A brief discussion about this somewhere in the text would be useful.

We suspect this is due to two factors; 1) unlike in the empirical data, in the model there is much less noise and behavioural variability, such that for strong enough inputs the model will achieve ceiling-level performance; 2) In the original manuscript we used neurometric functions to quantify the behaviour of the model, whereas psychometric functions were used in the empirical data. To make the model and data more directly comparable, in the revised manuscript we now fit the same psychometric function used by (Takahashi et al., 2016b, 2020) under the assumption that the model uses the optimal criterion (the criterion that best minimises misses and false alarms). This new method results in model psychometric functions that provide a better qualitative fit to the empirical data (see new figure below). We have also noted the differences due to behavioural variability in the manuscript:

P9: "We note that the saturating detection probability values in the model are due to an absense of behavioural stochasticity resulting from extranious factors such as decision noise which are inherent to empirical data."

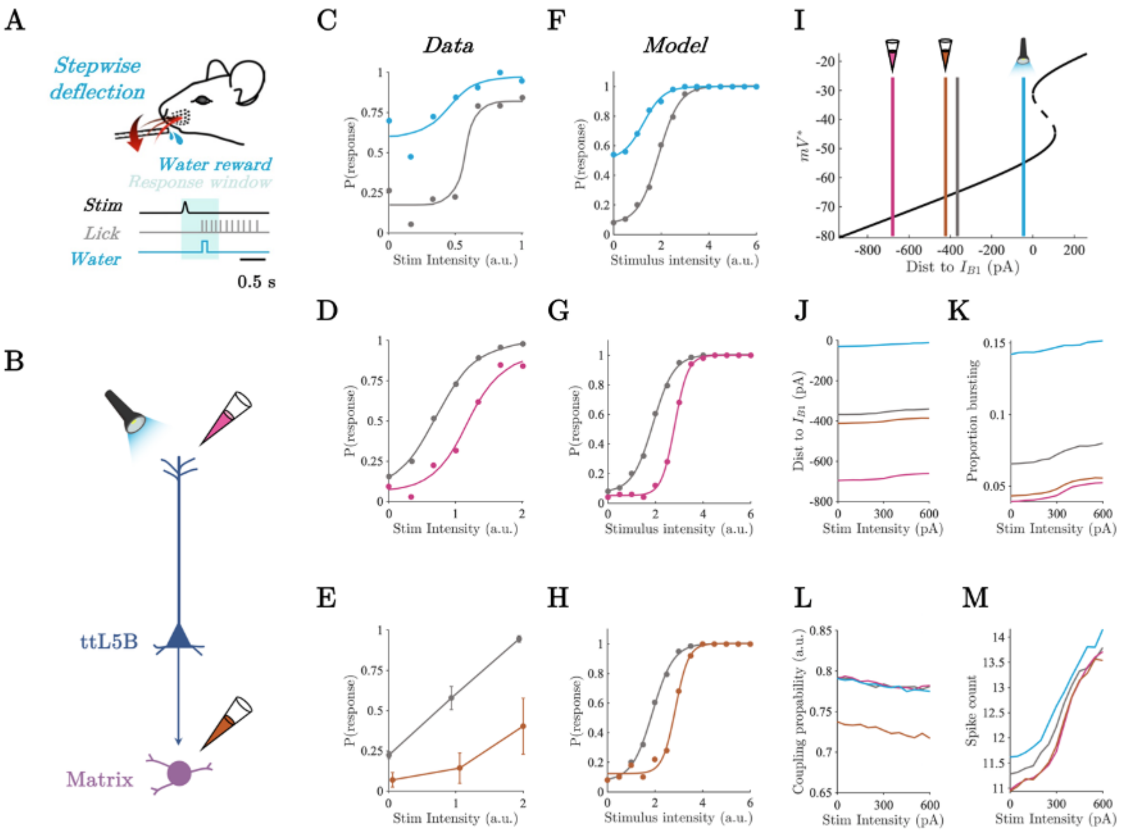

Fig 2. Apical compartment distance to bifurcation and thalamic-gating explains shifts in perceptual threshold. A) Whisker deflection paradigm modified from Takahashi et al, (2020). **B)** Representation of causal perturbations to $L5_{PT}$ thalamocortical circuit including optogenetic excitation of the apical dendrites (light blue), pharmacological inhibition of the apical dendrites (pink), and pharmacological

6) It might be interesting to test whether the visual rivalry results change in the model for stimuli not fully orthogonal. Is the statistics of dominance durations maintained? At which point (i.e. proximity between both stimuli) the bistable competition breaks down? This could provide exciting predictions for future psychophysics experiments, and would be easy to test in the model.

Response: We agree enthusiastically that simulating visual rivalry with non-orthogonal inputs is an interesting question to explore. Empirically, there is evidence showing that as the visual angle between gratings decreases the rate of rivalry slows (Andrews & Purves, 1997). This has been modelled previously (Laing & Chow, 2002) with a ring model of visual rivalry very similar to our own. In the model, the slowing effect is due to inhibition having broader projections than excitation leading to an additive effect of inhibition but not excitation slowing rivalry by reducing the rate at which the hyperpolarising adaptation current accumulates. We are currently working on a separate paper using a similar model that follows up on this finding in more detail. As the mechanism of slowing is only indirectly related to the burst-dependent corticothalamic mechanism of perceptual dominance we interrogate in this paper we would prefer to save exploration of the slowing effect for a future manuscript (which we are currently working on). For the

reviewers' interest, we have attached a basic figure from the model we are currently developing which explains the slowing effect of increasing visual similarity in terms of the additive effect of inhibition.

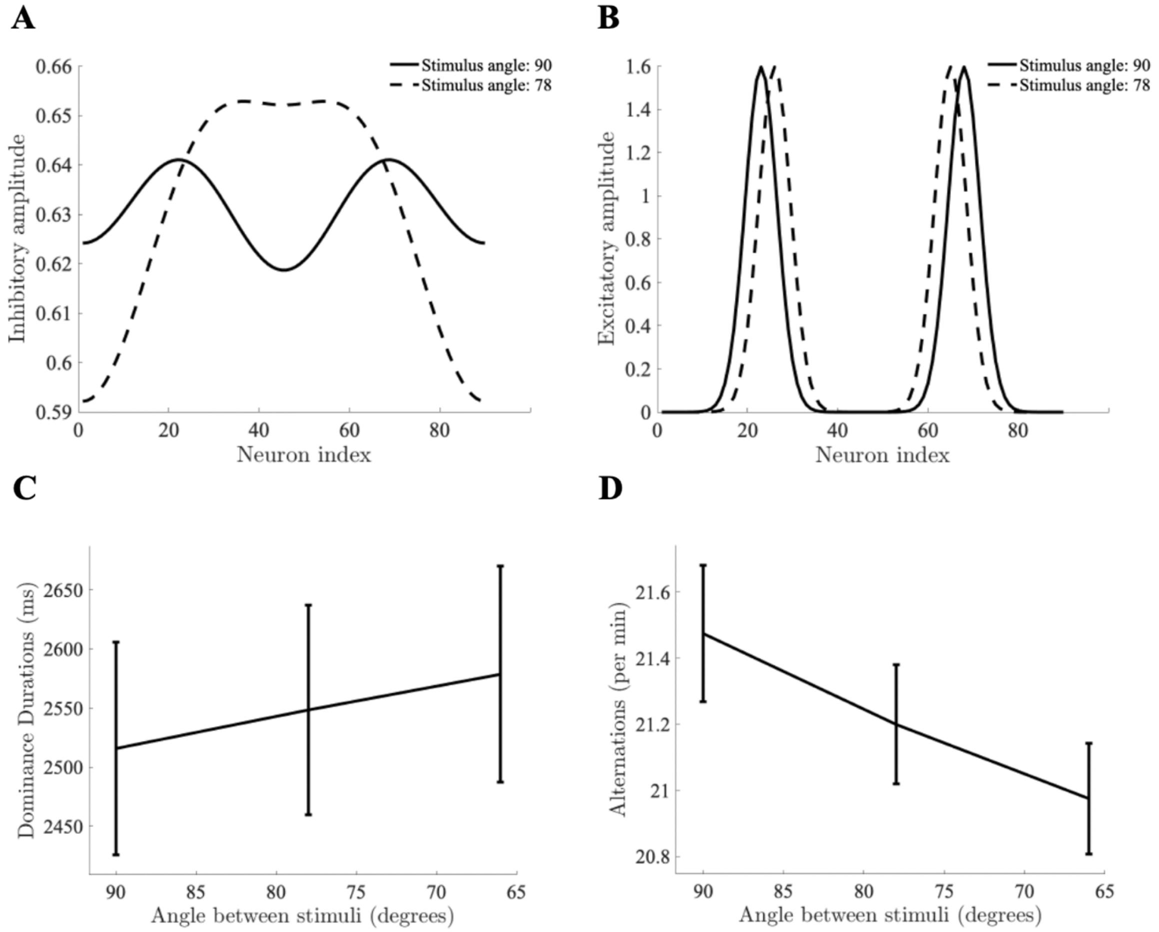

RR Figure. Rivalry slows as a function of visual angle between gratings. A) Amplitude of inhibitory to excitatory coupling for grating stimuli 90 (solid line) and 78 degrees apart (dashed line). **B)** Same as **A** but amplitude of excitatory coupling. **C)** Dominance duration as a function of angle between grating stimuli. **D)** Perceptual alternation frequency as a function of angle between grating stimuli.

References

Andrews, T. J., & Purves, D. (1997). Similarities in normal and binocularly rivalrous viewing. *Proceedings of the National Academy of Sciences*, *94*(18), 9905–9908. https://doi.org/10.1073/pnas.94.18.9905

Aru, J., Suzuki, M., & Larkum, M. E. (2020). Cellular Mechanisms of Conscious Processing. *Trends in Cognitive Sciences*, *24*(10), 814–825. https://doi.org/10.1016/j.tics.2020.07.006

Aru, J., Suzuki, M., Rutiku, R., Larkum, M. E., & Bachmann, T. (2019). Coupling the State and Contents of Consciousness. *Frontiers in Systems Neuroscience*, *13*, 43. https://doi.org/10.3389/fnsys.2019.00043

Bogatova, D., Smirnakis, S. M., & Palagina, G. (2024). Tug-of-Peace: Visual Rivalry and Atypical Visual Motion Processing in MECP2 Duplication Syndrome of Autism. *Eneuro*, *11*(1), ENEURO.0102-23.2023. https://doi.org/10.1523/ENEURO.0102-23.2023

Bonjean, M., Baker, T., Bazhenov, M., Cash, S., Halgren, E., & Sejnowski, T. (2012). Interactions between Core and Matrix Thalamocortical Projections in Human Sleep Spindle Synchronization. *Journal of Neuroscience*, *32*(15), 5250–5263. https://doi.org/10.1523/JNEUROSCI.6141-11.2012

Brascamp, J. W., Klink, P. C., & Levelt, W. J. M. (2015). The 'laws' of binocular rivalry: 50 years of Levelt's propositions. *Vision Research*, *109*, 20–37. https://doi.org/10.1016/j.visres.2015.02.019

Clascá, F., Porrero, C., Galazo, M. J., Rubio-Garrido, P., & Evangelio, M. (2016). Anatomy and Development of Multispecific Thalamocortical Axons. In *Axons and Brain Architecture* (pp. 69–92). Elsevier. https://doi.org/10.1016/B978-0-12-801393-9.00004-9

Clascá, F., Rubio-Garrido, P., & Jabaudon, D. (2012). Unveiling the diversity of thalamocortical neuron subtypes. *The European Journal of Neuroscience*, *35*(10), 1524–1532. https://doi.org/10.1111/j.1460-9568.2012.08033.x

Douglas, R. J., & Martin, K. A. C. (2004). Neuronal Circuits of the Neocortex. *Annual Review of Neuroscience*, *27*(1), 419–451. https://doi.org/10.1146/annurev.neuro.27.070203.144152

Harris, K. D., & Shepherd, G. M. G. (2015). The neocortical circuit: Themes and variations. *Nature Neuroscience*, *18*(2), 170–181. https://doi.org/10.1038/nn.3917

Hupé, J.-M., Signorelli, C. M., & Alais, D. (2019). Two paradigms of bistable plaid motion reveal independent mutual inhibition processes. *Journal of Vision*, *19*(4), 5. https://doi.org/10.1167/19.4.5

Jones, E. G. (2001). The thalamic matrix and thalamocortical synchrony. *Trends in Neurosciences*, *24*(10), 595–601. https://doi.org/10.1016/S0166-2236(00)01922-6

Jones, E. G. (2002). Thalamic circuitry and thalamocortical synchrony. *Philosophical Transactions of the Royal Society of London. Series B: Biological Sciences*, *357*(1428), 1659–1673. https://doi.org/10.1098/rstb.2002.1168

Jones, E. G., & Hendry, S. H. C. (1989). Differential Calcium Binding Protein Immunoreactivity Distinguishes Classes of Relay Neurons in Monkey Thalamic Nuclei. *European Journal of Neuroscience*, *1*(3), 222–246. https://doi.org/10.1111/j.1460-9568.1989.tb00791.x

Laing, C. R., & Chow, C. C. (2002). A Spiking Neuron Model for Binocular Rivalry. *Journal of Computational Neuroscience*, *12*(1), 39–53. https://doi.org/10.1023/A:1014942129705

Münkle, M. C., Waldvogel, H. J., & Faull, R. L. (2000). The distribution of calbindin, calretinin and parvalbumin immunoreactivity in the human thalamus. *Journal of Chemical Neuroanatomy*, *19*(3), 155–173. https://doi.org/10.1016/s0891-0618(00)00060-0

Palagina, G., Meyer, J. F., & Smirnakis, S. M. (2017). Complex Visual Motion Representation in Mouse Area V1. *The Journal of Neuroscience*, *37*(1), 164–183. https://doi.org/10.1523/JNEUROSCI.0997-16.2017

Poort, J., & Meyer, A. F. (2021). Vision: Depth perception in climbing mice. *Current Biology*, *31*(10), R486–R488. https://doi.org/10.1016/j.cub.2021.03.066

Qian, C., Chen, Z., Hollander, G. de, Knapen, T., Zhang, Z., He, S., & Zhang, P. (2023). *Hierarchical and fine-scale mechanisms of binocular rivalry for conscious perception* (p. 2023.02.11.528110). bioRxiv. https://doi.org/10.1101/2023.02.11.528110

Schulz, J. M., Kay, J. W., Bischofberger, J., & Larkum, M. E. (2021). GABAB Receptor-Mediated Regulation of Dendro-Somatic Synergy in Layer 5 Pyramidal Neurons. *Frontiers in Cellular Neuroscience*, *15*, 718413. https://doi.org/10.3389/fncel.2021.718413

Shepherd, G. M. G., & Yamawaki, N. (2021). Untangling the cortico-thalamo-cortical loop: Cellular pieces of a knotty circuit puzzle. *Nature Reviews Neuroscience*, *22*(7), 389–406. https://doi.org/10.1038/s41583-021-00459-3

Shpiro, A., Curtu, R., Rinzel, J., & Rubin, N. (2007). Dynamical Characteristics Common to Neuronal Competition Models. *Journal of Neurophysiology*, *97*(1), 462–473. https://doi.org/10.1152/jn.00604.2006

Storm, J. F., Klink, P. C., Aru, J., Senn, W., Goebel, R., Pigorini, A., Avanzini, P., Vanduffel, W., Roelfsema, P. R., Massimini, M., Larkum, M. E., & Pennartz, C. M. A. (2024). An integrative, multiscale view on neural theories of consciousness. *Neuron*, *112*(10), 1531–1552. https://doi.org/10.1016/j.neuron.2024.02.004

Strogatz, S. (2018). *Nonlinear dynamics and chaos: With applications to physics, biology, chemistry, and engineering* (Second edition). CRC Press Taylor & Francis Group.

Suzuki, M., & Larkum, M. E. (2020). General Anesthesia Decouples Cortical Pyramidal Neurons. *Cell*, *180*(4), 666-676.e13. https://doi.org/10.1016/j.cell.2020.01.024

Takahashi, N., Ebner, C., Sigl-Glöckner, J., Moberg, S., Nierwetberg, S., & Larkum, M. E. (2020). Active dendritic currents gate descending cortical outputs in perception. *Nature Neuroscience*, *23*(10), 1277–1285. https://doi.org/10.1038/s41593-020-0677-8

Takahashi, N., Oertner, T. G., Hegemann, P., & Larkum, M. E. (2016a). Active cortical dendrites modulate perception. *Science*, *354*(6319), 1587–1590. https://doi.org/10.1126/science.aah6066

Takahashi, N., Oertner, T. G., Hegemann, P., & Larkum, M. E. (2016b). Active cortical dendrites modulate perception. *Science*, *354*(6319), 1587–1590. https://doi.org/10.1126/science.aah6066

Wang, Z., Dai, W., & McLaughlin, D. W. (2020). Ring models of binocular rivalry and fusion. *Journal of Computational Neuroscience*, *48*(2), 193–211. https://doi.org/10.1007/s10827-020-00744-7

Wilson, H. R. (2003). Computational evidence for a rivalry hierarchy in vision. *Proceedings of the National Academy of Sciences*, *100*(24), 14499–14503. https://doi.org/10.1073/pnas.2333622100

Yazdanbakhsh, A., Barbas, H., & Zikopoulos, B. (2023). Sleep spindles in primates: Modeling the effects of distinct laminar thalamocortical connectivity in core, matrix, and reticular thalamic circuits. *Network Neuroscience*, *7*(2), 743–768. https://doi.org/10.1162/netn_a_00311
