# Supplementary material for: A burst-dependent thalamocortical substrate for perceptual awareness
Source: PLoS Comput Biol. 2025 Apr 7;21(4):e1012951. doi: 10.1371/journal.pcbi.1012951 (PMC12061433; doi:10.1371/journal.pcbi.1012951)
Supplement: Attachment — Submitted filename: PCB_RR2.docx [file pcbi.1012951.s004.docx]

**We thank the reviewers for the time and consideration of the first round of responses. Here we address the 3 remaining comments of reviewer 1.**

**Comment 1:**

Regarding Fig 3F: I must be misreading the figure. If I look at the mean values, the solid line goes from 3.5 to 5 and the dashed from 3.5 to 1.5, so that this in fact contradicts the second principle as (5-3.5) < (3.5-1.5).

Of course if you take the highest value of the std dev bar for the solid and the highest for the dashed, the you obtain what the authors say here, but that seems a little unfair and the means tell a quite different story

**This is a mistake on our end and an excellent catch by the reviewer. In order simulate our scaled up model for the last round of reviews we had to make the code base compatible with our high powered computing resources. Having done this, we took the opportunity increase the robustness of our results by rerunning all of the simulations across more initialisations. However, we also introduced an error into parameter values of the simulations of Levelt’s second law and started the stimulus strength sweep too low (1350Hz) for the fixed strength stimulus which meant that the strength of the competing stimulus reduced to 1200Hz. To give some context for these values, in our simulation of Levelt’s fourth law 1300Hz of external drive is the switching point where dominance durations speed up rather than slow down. We have now rerun the Levelt’s second law simulations starting the fixed stimulus at 1400 Hz and have run the simulation across more random seeds to ensure the validity of the results. The simulation results now agree with Levelt’s second law (i.e. 6.5-3 > 3-1). The new figure is shown below. We have also double checked the parameter values and code for the other simulations to ensure that we did not introduce any other mistakes.**

**Comment 2:**

The authors emphasize the burst dependent mechanisms in perception. So, I think they should run some rivalry simulations when they reduce the bursting for example, by gradually decoupling the soma and dendrite. Most other models of BR dont need bursting so I remain skeptical of why this is necessary. Some of this is sort of done in Fig 5, but I'd like a more systematic study

**This is an excellent suggestion. In section 3 of the supplementary material we have modified the sweep of the parameters controlling the reset conditions of each L5PT cell to now include a sweep of the apical to somatic (and v.v.) coupling probability which is set to a specific stationary value for each simulation instead of being controlled dynamically by projections from the thalamic population. The results of the parameter sweep (shown below), show that a minimum coupling probability of ~0.9 is necessary in order for the model to maintain dominance durations in the range typical of rivalry (i.e. with a period on the order of seconds) across a wide range of parameter values. When the coupling is dynamical and dependent on thalamic conductances (shown in A below) typical coupling probability values are ~ 0.98 for the dominant population and ~ 0.1 for the suppressed population (see Fig 3D in the paper).**

**We have also included an additional point of clarification in the discussion section on the role of bursting in our model and the relationship to previous models of rivalry (page 23).**

***“Importantly, the simulation of these experimental perturbations would not be possible in any existing models of rivalry, even those at the spiking level (e.g. Laing & Chow, 2002; Wang et al., 2020; Wilson, 2003), as they focus on the minimal conditions for rivalry in point-neuron models of cortical interaction. The inclusion of a dual compartment model of L5_PT_ cells, and an explicit thalamic population, was, therefore, required in order to make contact with the results of Takahashi et al (2016, 2020). Indeed, although (thalamus-gated) plateau potential induced bursting is necessary for rivalry in our model, if we were to coarse grain the model the dynamics would be well described by a mean field model tracking the mean firing rate of each population. Thalamic controlled L5_PT_ bursting is therefore not strictly speaking necessary for modelling the mesoscale dynamics of binocular rivalry, but it is necessary if one wants to capture the neurobiological processes that underpin the mesoscale dynamics and how cellular level interventions such as optogenetic excitation or pharmacological inhibition would impact rivalry dynamics as we have done here.”***

**Comment 3:**

Please replace "whilst" with "while"

**Done**
